# Multiscale CNN-state space model with feature fusion for crop disease detection from UAV imagery

**DOI:** 10.3389/fpls.2025.1733727

**Published:** 2025-12-17

**Authors:** Ting Zhang, Dengwu Wang, Wen Chen

**Affiliations:** School of Computer Science, Xijing University, Xi’an, China

**Keywords:** crop disease detection, superpixel segmentation, unmanned aerial vehicle (UAV), Visual StateSpace (VSS), multiscale CNN-VSS with feature fusion (MSCNN-VSS)

## Abstract

Accurate detection of crop diseases from unmanned aerial vehicle (UAV) imagery is critical for precision agriculture. This task remains challenging due to the complex backgrounds, variable scales of lesions, and the need to model both fine-grained spot details and long-range spatial dependencies within large field scenes. To address these issues, this paper proposes a novel Multiscale CNNState Space Model with Feature Fusion (MSCNN-VSS). The model is specifically designed to hierarchically extract and integrate multi-level features for UAVbased analysis: a dilated multi-scale Inception module is introduced to capture diverse local lesion patterns across different scales without sacrificing spatial detail; a Visual State Space (VSS) block serves as the core component to efficiently model global contextual relationships across the canopy with linear computational complexity, effectively overcoming the limitations of Transformers on high-resolution UAV images; and a hybrid attention module is subsequently applied to refine the fused features and accentuate subtle diseased regions. Extensive experiments on a UAV-based crop disease dataset demonstrate that MSCNN-VSS achieves state-of-the-art performance, with a Pixel Accuracy (PA) of 0.9421 and a mean Intersection over Union (mIoU) of 0.9152, significantly outperforming existing CNN and Transformer-based benchmarks. This work provides a balanced and effective solution for automated crop disease detection in practical agricultural scenarios.

## 1 Introduction

Global crop production is seriously threatened by various diseases such as aphids, powdery mildew and yellow rust, causing huge economic losses (Abbas et al., 2023; Zhang et al., 2023; Jia et al., 2025). Image segmentation of diseased leaves of crops is the key to disease detection and prevention (Xu et al., 2022a, 2022; Zhang et al., 2023). Unmanned aerial vehicle (UAV) remote sensing has become an important technical means for the detection and identification of large-scale crop diseases (Bouguettaya et al., 2023; Li et al., 2024; Shahi et al., 2023). It possesses obvious advantages, including high spatial resolution, operational flexibility, efficiency, and the ability to conduct rapid and low-cost monitoring of large areas under the conditions of high reliability and high data resolution (Maes and Steppe, 2019). The accurate detection of diseases from UAV imagery relies on advanced analytical methods. The field has evolved from traditional machine learning towards deep learning. Convolutional Neural Networks (CNNs) and U-Net architectures have demonstrated remarkable performance by automating feature learning (Qin et al., 2021; Kerkech et al., 2020; Zhu et al., 2024; Zhang and Zhang, 2023). However, these models are inherently constrained by their limited receptive field, making it difficult to capture long-range dependencies and global contexts in crop disease images (Lei et al., 2021). This limits their ability to model correlations between distant lesions.

To overcome the receptive field limitation, Transformer-based models were introduced. They demonstrate the capability to handle inputs of varying dimensions and dynamically extract critical information through self-attention (De and Brown, 2023). Transformers can capture complex spatial dependency relationships between leaf lesions and healthy tissues, effectively identifying subtle spectral features of early diseases even under complex backgrounds (Singh et al., 2024). Frameworks like PD-TR (Wang J, et al., 2024) show significant advantages in cross-regional lesion correlation modeling. However, due to their quadratic complexity, Transformers impose a significant computational cost when processing high-resolution and high-dimensional UAV images.

Recently, Visual State Space (VSS) models have aroused great interest as an efficient alternative (Alonso et al., 2024). SSM-based Mamba models have shown great potential for long-range dependency modeling with linear complexity (Hu et al., 2024). By employing a state space mechanism, VSS models offer a more balanced approach for modeling both global contexts and local features in UAV crop disease detection. Architectures like VM-UNet (Ruan et al., 2024), Swin-UMamba (Shi et al., 2024), and Multiscale Vision Mamba-UNet (MSVM-UNet) (Hu et al., 2024) have demonstrated the potential of VSS blocks in segmentation tasks. Despite these promising developments, the potential of SSM and VSS architectures has rarely been fully exploited in UAV crop disease detection (Bouguettaya et al., 2023; Narmilan et al., 2022).

To bridge this gap, this paper constructs a multiscale CNN-VSS with feature fusion (MSCNN-VSS) for crop disease detection. The main contributions are summarized as follows:

1. A hybrid CNN-VSS architecture is constructed, providing a balanced solution for the segmentation of complex unmanned aerial vehicle (UAV) crop disease images.
2. Collaboratively integrate multi-scale convolutional, VSS and hybrid attention modules, to enhance local feature diversity, capture global context dependencies, and optimize feature representation.
3. Extensive experiments were conducted on the dataset of crop disease images based on unmanned aerial vehicles.

The rest of this paper is arranged as follows. Section 2 introduces the proposed MSCNN-VSS, focusing on its main module design. Section 3 presents the experimental setup, benchmark results, and a comprehensive analysis including ablation studies and visualizations for welding defect segmentation. Finally, Section 4 concludes this paper.

## 2 The MSCNN-VSS model

**Figure 1:**
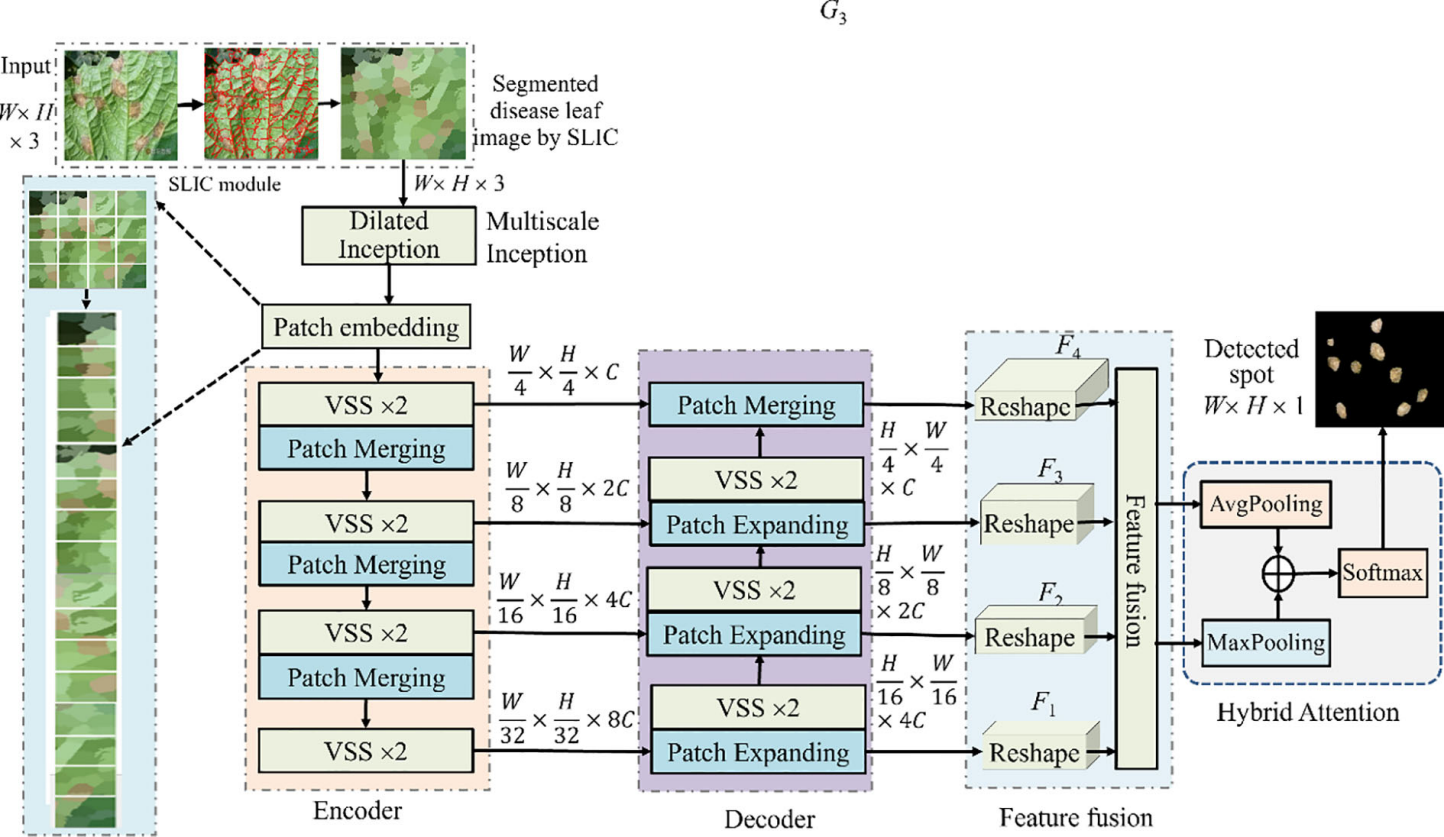
Architecture of MSCNN-VSS.

**Figure 2:**
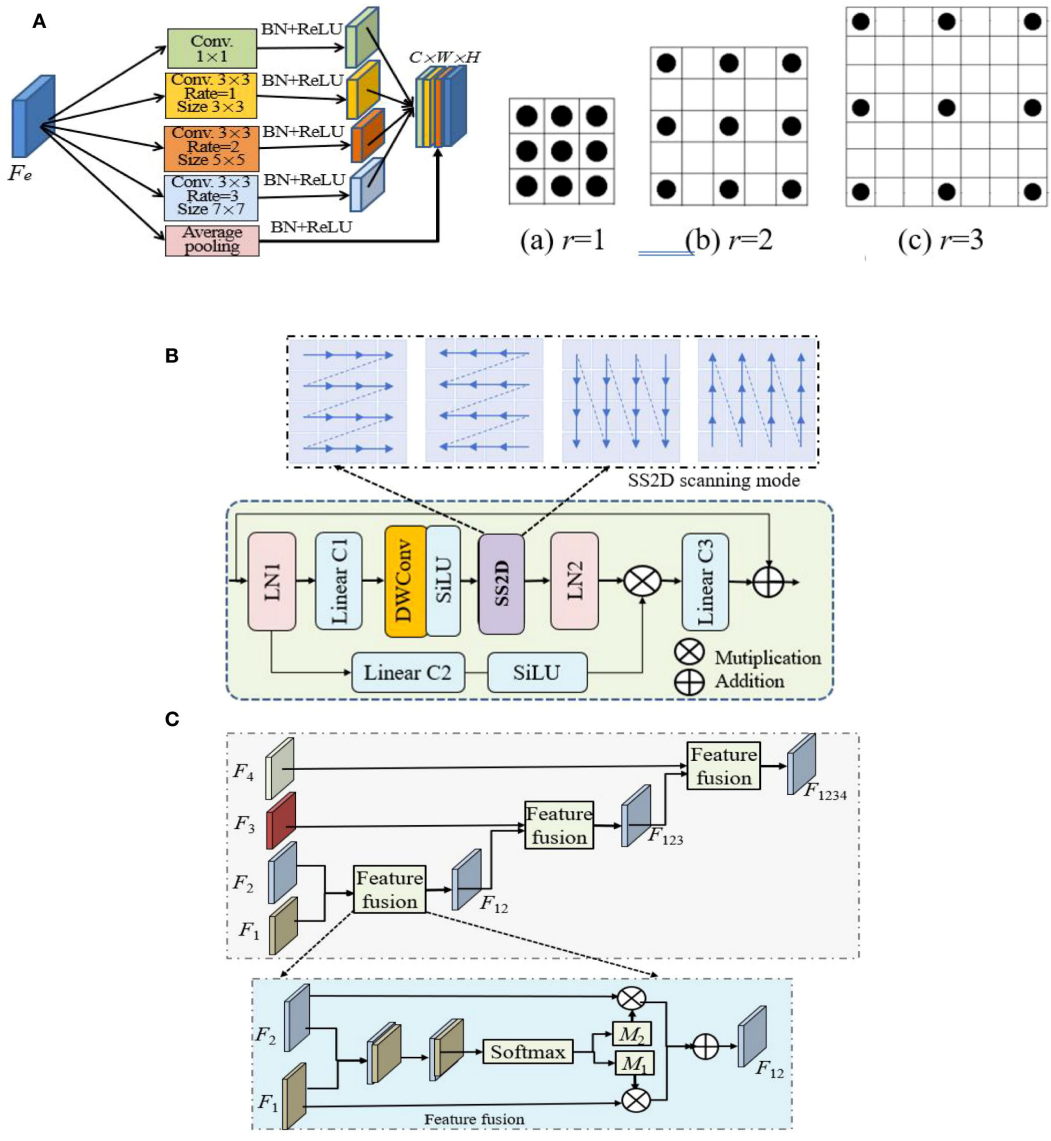
The structures of three main modules, where ⊕ and ⊗ indicate add and Hadamard product operations. **(A)** Multiscale Inception and its 3 dilated kernels (r = 1,2,3). **(B)** VSS. **(C)** Feature fusion.

The architecture of MSCNN-VSS is shown in **Figure 1**, consisting of Superpixel block, encoder, decoder, feature fusion module, and hybrid attention, where encoder and decoder are combined by skip connection to achieve better segmentation results with few annotation images. The main structures are shown in **Figure 2**, where VSS block derived from VMamba is the backbone of encoder and decoder, the dimension of the input image is *W×H*×3, and the output channel of encoder is 2*^i^*×*C*, *C* is often set to 96, and the output channel of decoder is the opposite of that of encoder, gradually decreasing.

The notation and description of key mathematical symbols and operations are shown in **Table 1**.

**Table 1:** Notation and description of key mathematical symbols and operations.

Symbol/Operation	Description	Symbol/Operation	Description
*X*	Input feature map to a block or operation.	Atten(·)	Spatial attention mechanism block.
*Z*	Output feature map of a block or operation.	*Ai*2​	Attention maps adaptively learned by the scale-aware block for feature *Fi*​.
*Y*	Final predicted segmentation map.	*H*attention	Output feature map from the hybrid attention module.
*Y*^	Ground truth annotation (label).	AP(·)	Global Average Pooling operation.
VSS Block		MP(·)	Global Max Pooling operation.
*X*′	Intermediate feature map after the first linear projection and layer normalization.	*σ*(·)	Sigmoid activation function.
*X*′′	Intermediate feature map after depthwise convolution and SiLU activation.	L	Total loss (Binary Cross-Entropy).
*X*^	Intermediate feature map prepared for the SS2D operation.	*C*	Number of classes (*C* = 2 for binary segmentation: disease vs. background).
LN(·)	Layer Normalization.	*yi*​	True label of the *i*-th pixel.
Lin(·)	Linear projection layer (implemented as a 1×1 convolution).	*y*^​*i*​	Predicted probability of the *i*-th pixel belonging to the disease class.
SiLU(·)	Sigmoid Linear Unit activation function.	*yi*​=*c*​	Indicator function that is 1 if the true label *yi*​ equals class *c*, else 0.
DWConv(·)	Depthwise Convolution.	⊙	Element-wise multiplication (Hadamard product).
SS2D(·)	2D Selective Scan operation, the core mechanism for long-range dependency modeling.	⊕	Element-wise addition.
*Fi*​	The *i*-th feature map from the decoder, where *i*∈{1,2,3,4}.	∥	Concatenation operation along the channel dimension.
*V*12​, *V*123​,*V*1234​	Fused feature maps at different hierarchical levels.		

The main processes of MSCNN-VSS are introduced in detail as follows.

1. Superpixel segmentation is performed using the Simple Linear Iterative Clustering (SLIC) algorithm from the OpenCV-Python library. We test different numbers of superpixels on images of diseased leaves (both close-up and distant views).

2. Segmenting diseased leaves in UAV imagery is challenging due to the high variability in lesion appearance. The pooling layers in standard U-Ne lose spatial detail, harming lesion localization. Therefore, a multi-scale dilated Inception module is introduced. Its structure is shown in **Figure 2A**, which is a series of parallel 3×3 convolution with dilated rates of {1,2,3}, exponentially increasing the receptive field (from 3×3 to 7×7) without increasing the weight parameters. After each dilated convolution, batch normalization (BN) and ReLU, the branched image feature details are concatenated and aggregated through 1×1 convolution to speed up the network training and convergence.

3. Encoder. Following patch embedding, the transformed features from the dilated Inception module are input into the encoder. The encoder is composed of VSS×2 blocks, whose fundamental operation is the 2D-selective-scan (SS2D). VSS is designed to overcome the limitations of standard models in capturing long-range dependencies in 2D imagery. Its architecture is depicted in **Figure 2B** and is detailed as show in Equation 1:

(1) $$\begin{array}{l} V_{out}=VSS(V_{in})=Lin3(V_{1}⊗V_{2})\\ V_{1}=LN(SS2DM(SiLU(DWConv(Lin1(LN(V_{in}))))\\ V_{2}=SiLU(Lin2(LN(V_{in}))) \end{array}$$

where $V_{in},V_{out}$ are the input and output feature maps of MSVSS, *LN*(·), *Lin*(·), *SiLU*(·), *MSSSM*(·) and *DWConv*(·) are layer normalization, linear projecting, SiLU activation, MSSSM and DWConv operations, respectively.

SSM adopts the multiscan strategy to model long-range feature dependencies, which significantly increases the feature redundancy. Patch merging for 2× down-sampling in the encoder captures long-range dependencies while gradually reducing the spatial dimension, effectively compressing the input into multiscale representations.

4. Decoder. Like encoder, decoder consists of VSS and patch expanding blocks, where patch expanding is 2×up-sampling operation.

5. Feature fusion. Feature fusion is leveraged to integrate multi-scale features. It is commonly used in various deep learning models. Its structure is shown in **Figure 2C**. To manage the computational complexity, 1×1 convolution reshapes the feature mapping and standardizes the number of channels for decoder features to 64 at all scales. The module hierarchically integrates these multi-scale features using a spatial attention mechanism between adjacent scales. The integrated features from one level interact iteratively with those of the next, enabling adaptive multi-scale fusion. This process is described as show in Equation 2:

(2) $$\begin{array}{l} V_{12}=Atten(F_{1},F_{2}),\;\\ V_{123}=Atten(F_{12},F_{3}),\;\\ V_{1234}=Atten(F_{123},F_{4}) \end{array}$$

where *Atten*(.) denotes attention mechanism block, *F_i_*, *i*∈{1, 2, 3, 4} are the *i*th features generated by the decoder and have been upsampled, with the same resolution, but different numbers of channels.

Taking *V*_12_ as an example, show in Equation 3:

(3) $$\begin{array}{l} V_{12}=Atten(F_{1},F_{2})\\ \;\;\;\;\;=M_{1}⊗F_{1}+M_{2}⊗F_{2} \end{array}$$

where $M_{1}$ and $M_{2}$ are the attention maps adaptively learned by the scale-aware block, $F_{1},F_{2}$ are concatenated and input into convolution and Softmax layers, the output is split along the channel dimension to obtain $M_{1}$ and $M_{2}$.

(6) Hybrid attention. The integrated fusion feature *V*_1234_ is input into hybrid attention along the spatial dimension to aggregate the spatial information, generating two 1D average pooling and maxpooling maps, which are concatenated. Hybrid attention can enhance feature representation of the model, which is simply described as follows,

(4) $$HAtten=Avgpooling(V_{1234})⊕Maxpooling(V_{1234})$$

where Avgpooling(.) and Maxpooling(.) are the average pooling and maxpooling operations, respectively.

(7) Training model. Softmax classifier is used to detect crop diseases by *Hatten* from Equation 4. Classification binary cross entropy objective function is employed to measure the loss between the actual and the predicted detection distribution, defined as show in Equation 5:

(5) $$J(W)=-\frac{1}{N}\sum_{n=1}^{N}\sum_{c=1}^{C}\ell (y_{n}==c)\log(\exp(W_{c}^{T}X_{i})/\sum_{p=1}^{C}\exp(W_{p}^{T}X_{i}))$$

where $\left(X_{i},y_{i}\right)$(*i* = 1,2,…,*N*) is a training image, $X_{i}$ is the *i*th pixel, $W_{c}^{T}X_{i}$ is the feature representation of $X_{i}$, $y_{i}$ is its corresponding label, *N* and *C* are the numbers of the pixel and corresponding class in the image, respectively, l(*) is the index function, *C* = 2 represents the detected result is binary image, containing defect pixel and background pixel.

**Figure 3:**
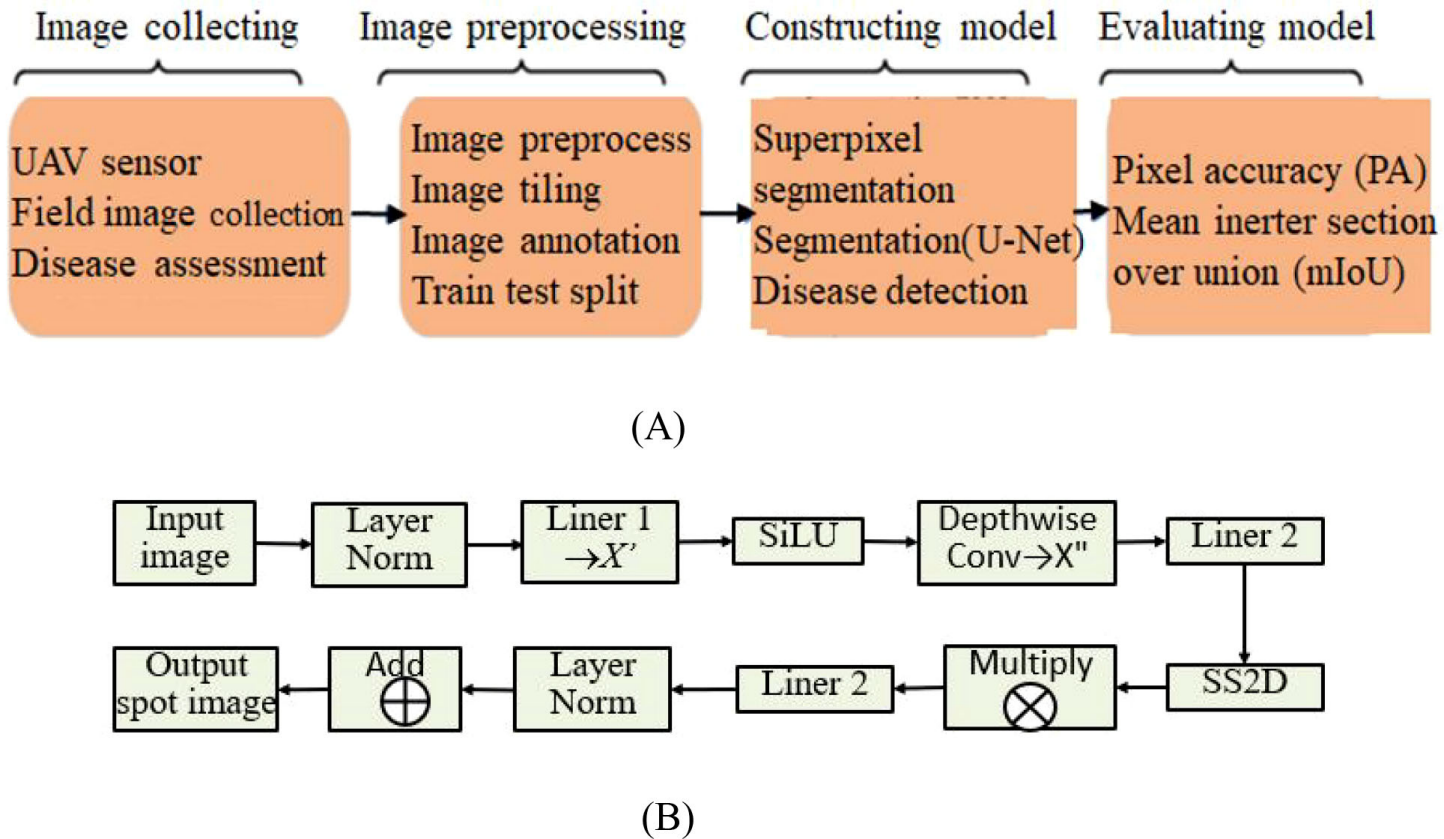
The general process of MSCNN-VSS based crop disease detection using UAV imagery. **(A)** Stages. **(B)** Steps.

From the above analysis, the problem of using MSCNN-VSS and UAV images for crop disease detection is described as shown in **Figure 3**, including four stages: collecting images, image preprocessing, constructing model, and evaluating model.

## 3 Experiment and analysis

The proposed MSCNN-VSS is extensively evaluated against six state-of-the-art models: Spatial-Context-Attention Network (SCANet) (Qin et al., 2021), an improved U-Net segmentation model with image processing (IUNet-IP) (Zhu et al., 2024), PD-TR (Wang H, et al., 2024), CMTNet (Guo et al., 2025), VM-UNet (Ruan et al., 2024), and Multiscale Vision Mamba-UNet (MSVM-UNet) (Hu et al., 2024). Brief descriptions of these comparative models are provided as follows.

SCANet is a spatial-context-attention network to identify disease based on UAV multi-spectral RSIs.

IUNet-IP is a hybrid architecture to identify leaf diseases and detect crops using deep learning and sophisticated image-processing techniques.

PD-TR is an end-to-end plant diseases detection using a Transformer.

CMTNet is a hybrid CNN-transformer network for UAV-based crop classification in precision agriculture.

VM-UNet is a Vision Mamba UNet for image segmentation.

MSVM-UNet is a multiscale VM-UNet for image segmentation.

### 3.1 UAV dataset preparation

**Figure 4:**
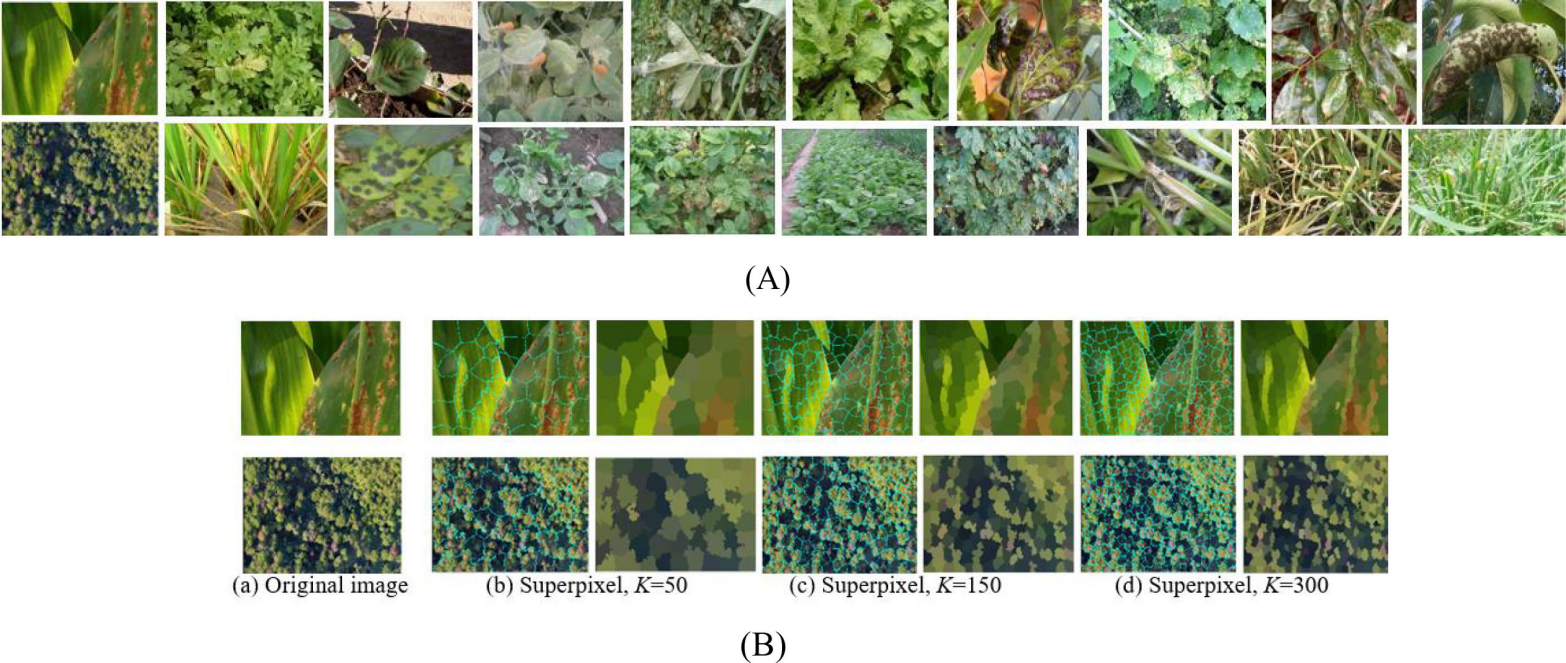
The rust diseased leaf image samples and superpixel images. **(A)** Diseased leaf image samples. **(B)** Superpixel images via three superpixel number.

Sensors and cameras installed on UAVs were used to capture images of crop diseased leaves. The experimental dataset was constructed using a commercial Spreading Wing S1000+ UAV equipped with an RGB sensor, which captured images of rust-diseased leaves across multiple crop species. Data acquisition was conducted from July to September 2022 in the Yangling Agricultural Demonstration Zone, Xi’an, China. The UAV was operated at a constant altitude of 25 meters, yielding imagery with a ground resolution of approximately 1 cm/pixel. Each image has a resolution of 4000×3000 pixels at 72 dpi. The dataset comprises 500 high-resolution images collected under varied scenarios, lighting conditions, shooting angles, and backgrounds, as illustrated in **Figure 4A**. To ensure robust evaluation, the dataset was constructed with a balanced distribution across five major crop species and three disease severity levels. The severity was labeled by agronomy experts based on the visible percentage of leaf area affected: ‘Small’ (1-10%), ‘Medium’ (11-25%), and ‘Large’ (>25%). The detailed distribution of samples is shown in **Table 2**.

**Table 2:** The detailed distribution of samples.

Crop species	Small	Medium	Large	Total
Maize	30	45	28	103
Wheat	29	42	25	96
Soybean	34	48	30	112
Rapeseed	27	39	24	90
Rice	31	41	27	99
Total	151	215	134	500

These samples cover maize, wheat, soybean, rapeseed, and rice 5 major crop species, with each crop category further divided into small, medium and large three severity levels. The detailed distribution is shown in Table below.

To ensure effective model training and evaluation, a five-fold cross-validation (FFCV) scheme was employed. The dataset was partitioned at the image level into five folds, ensuring that each fold maintained a nearly identical distribution of crop species and disease severity levels as the overall dataset (as shown in **Table 2**). This stratified splitting principle prevents bias and guarantees that each fold is representative of the entire data distribution. In each fold, 400 images (80%) were used for training, and the remaining 100 images (20%) served as an independent test set. The acquired images underwent preprocessing to eliminate background interference and noise. The Simple Linear Iterative Clustering (SLIC) method from the OpenCV-Python library was used for superpixel segmentation. We evaluated superpixel numbers in the range of [100, 300, 500]. Using 300 superpixels was selected as it preserved critical disease features without noticeable loss of spots while drastically reducing the computational load for subsequent analysis, as shown in **Figure 4B**. This setting was used for all experiments.

### 3.2 Implementation details

To ensure a fair and reproducible comparison among all models, we established a unified training benchmark with fixed hyperparameters, data augmentation, and input resolution across all compared methods.

Input Preprocessing: All UAV images were first processed by the SLIC superpixel algorithm (with 300 superpixels) and then centrally cropped to a uniform resolution of 512×512 pixels to ensure consistent input dimensions across the network.

Data Augmentation: To enhance model robustness and prevent overfitting, a standard set of augmentation strategies was applied on-the-fly during training to all models. This included: random horizontal and vertical flipping (probability=0.5), random rotation (± 30 degrees), and random adjustments to brightness and contrast (variation factor=0.2).

Training Configuration: All models were trained from scratch under the same conditions:

Epochs 300, Batch Size 15, Optimizer Adam, Initial Learning Rate 0.001, Learning Rate Schedule Reduced by 50% every 500 iterations, Loss Function Weighted Cross-Entropy.

The network parameters were initialized using the Kaiming method. All experiments were conducted in the hardware and software environments shown in **Table 3**.

**Table 3:** Experimental configuration of hardware and software.

Category	Specification
CPU	Intel Xeon E5-2667v @ 3.20 GHz
GPU	NVidia Quadro M4000
Operating System	Windows 10
Programming Language	Python 3.6
Deep Learning Framework	Keras, TensorFlow-GPU 1.8.0

In addition to PA and mIoU, the following evaluation metrics are used to assess the model, where quantifies the computational cost in the reasoning process, and the model size indicates the storage requirements.

Evaluation Metrics: Pixel Accuracy (PA), mean Intersection over Union (mIoU), parameters (Params), computational complexity (FLOPs), and training time. PA and mIoU are calculated as show in Equations 6 and 7:

(6) $$PA=\sum_{i=0}^{N}p_{ii}/\sum_{i=0}^{N}T_{i}$$

(7) $$mIoU=\frac{1}{N}\sum_{i=0}^{N}\frac{q_{ii}}{T_{i}+\sum_{j=0}^{N}\left(q_{ji}-q_{ii}\right)}$$

where *N* is the number of categories of image pixels, $p_{ii}$ is the predicted and actual pixel of type *i*, $T_{i}$ is the total number of class *i* pixels, $q_{ji}$ is the total number of pixels of actual type *i* and predicted type, *mIoU* is the mean Intersection over Union. The higher *mIoU* indicates the better match between the detected disease area and the actual disease area.

### 3.3 Experiment results

**Figure 5:**
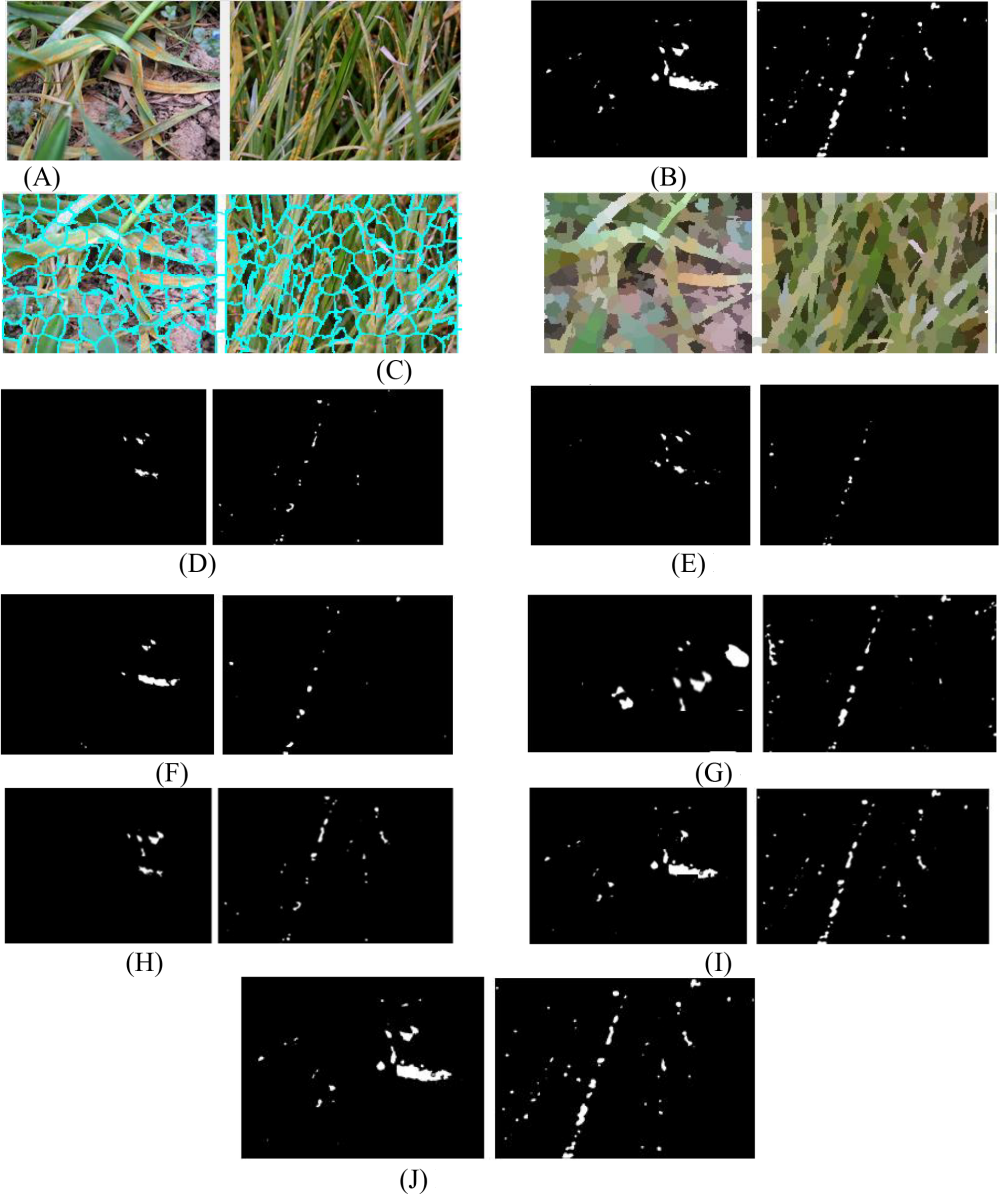
The detected spots of 7 models. **(A)** Original rice disease images. **(B)** Ground truth annotations. **(C)** Superpixel images. **(D)** SCANet. **(E)** IUNet-IP. **(F)** PD-TR. **(G)** CMTNet. **(H)** VM-UNet. **(I)** MSV-UNet. **(J)** MSCNN-VSS.

**Figures 5A and B** show two UAV-captured close-range images of rust-infected leaves and their corresponding annotated ground truths. **Figure 5C** displays the superpixel segmented results by SLIC, which divide the spot area into structurally meaningful components. Comparative segmentation results in **Figures 5D-J** demonstrate the performance of SCANet, IUNet-IP, PD-TR, CMTNet, VM-UNet, MSVM-UNet, and the proposed MSCNN-VSS, respectively.

**Figure 5** illustrates the segmentation performance of the compared models on two close-range UAV images of rust-infected leaves (**Figure 5A**) and their SLIC superpixel results (**Figure 5C**). While all models can detect disease spots under challenging conditions, MSCNN-VSS (**Figure 5J**) achieves the best results, identifying minute spots with minimal false positives due to its VSS block and feature fusion module, which enhance multi-scale feature representation. SCANet and IUNet-IP (**Figures 5D, E**) show significant false positives, while VM-UNet and MSVM-UNet (**Figures 5H, I**) capture broader lesions but lack boundary precision.

**Figure 6:**
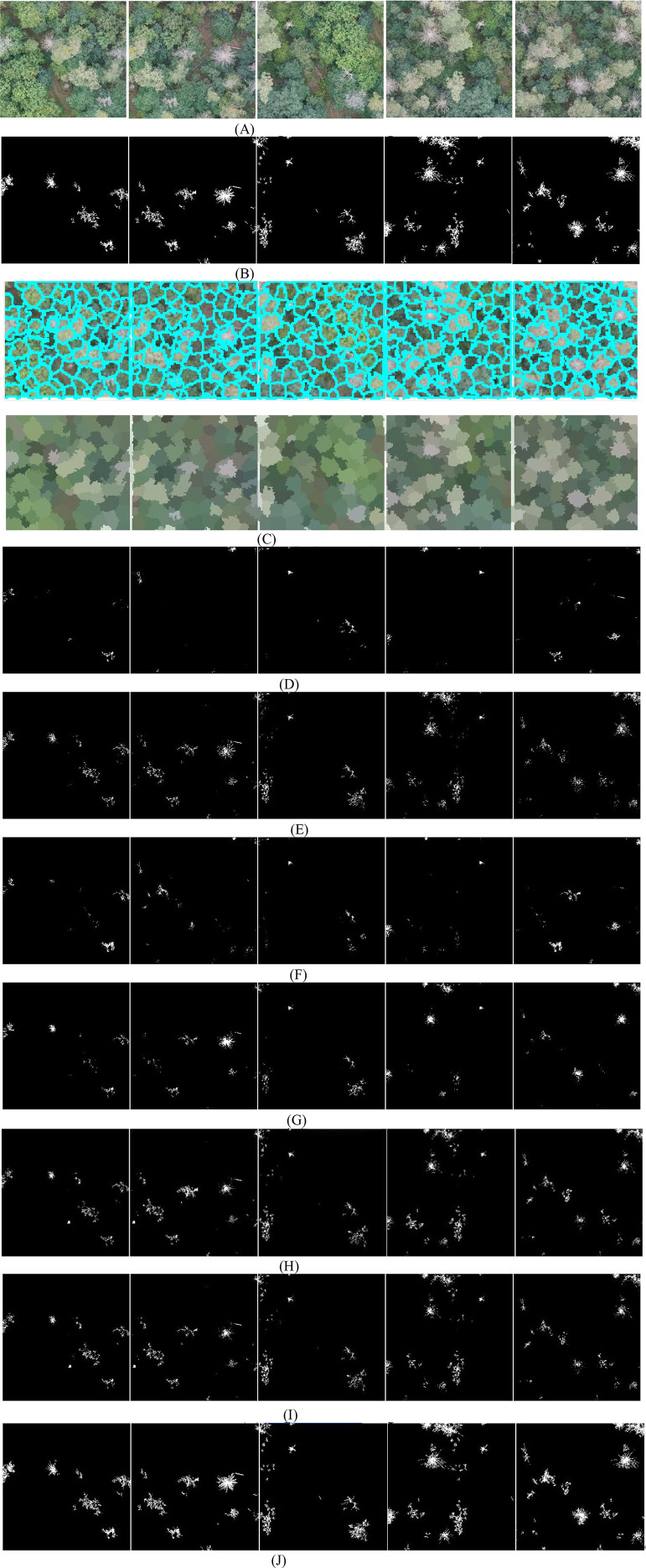
The detected spots of 7 models. **(A)** Original disease images. **(B)** Ground truth annotations. **(C)** Superpixel images. **(D)** SCANet. **(E)** IUNet-IP. **(F)** PD-TR. **(G)** CMTNet. **(H)** VM-UNet. **(I)** MSV-UNet. **(J)** MSCNN-VSS.

To further evaluate the performance of MSCNN-VSS, experiments are conducted on the diseased leaf images captured by UAVs at long distances. The comparative detection results of the 7 models are presented in **Figure 6**.

From **Figure 6**, it is found that on long-distance UAV images with complex backgrounds and weaker lesion characteristics, the performance of the 7 models shows significant differentiation, where SCANet and IUNet-IP generate a large number of background false detections and loss a lot of spots, PD-TR and CMTNet seriously miss detections of small lesions. Although VM-UNet and MSVM-UNet can maintain the basic structure, the boundary positioning is ambiguous. In contrast, the proposed MSCNN-VSS achieves the most complete capture and the complete boundary characterization of fine lesions while maintaining the lowest false detection rate. Its multi-level feature fusion mechanism and hybrid attention design effectively overcome the problem of feature attenuation in long-distance imaging.

To further validate the disease detection performance of the proposed model, the seven comparative models are evaluated using PA, mIoU, Parameters, FLOPs, Model size and average model-training time as objective metrics. Comparative experimental results (mean ± standard deviation) are shown in **Table 4**, where 95% Confidence Interval (95% CI) for PA and mIoU is based on the five-fold cross-validation.

**Table 4:** The different experimental set and results.

Results Model	PA	mIoU	PA 95% CI	mIoU 95% CI	Params (M)	FLOPs (G)	Size (MB)	Training Time (min)
SCANet	0.7198 ± 0.0045	0.6812 ± 0.0036	0.7198 ± 0.0056	0.6812 ± 0.0045	45.2	12.5	180.5	163.4
IUNet-IP	0.8186 ± 0.0057	0.7055 ± 0.0045	0.8186 ± 0.0071	0.7055 ± 0.0056	31.8	9.8	127.3	177.6
PD-TR	0.8409 ± 0.0014	0.7764 ± 0.0018	0.8409 ± 0.0018	0.7764 ± 0.0024	128.5	35.2	513.8	226.9
CMTNet	0.8763 ± 0.0040	0.8130 ± 0.0032	0.8763 ± 0.0050	0.8130 ± 0.0040	95.7	28.9	382.9	254.6
VM-UNet	0.8912 ± 0.0037	0.8423 ± 0.0030	0.8912 ± 0.0046	0.8423 ± 0.0037	25.1	7.5	100.5	113.3
MSVM-UNet	0.9152 ± 0.0036	0.8575 ± 0.0038	0.9152 ± 0.0045	0.8575 ± 0.0047	38.6	10.2	154.6	124.6
MSCNN-VSS	**0.9421 ± 0.0031**	**0.9152 ± 0.0036**	**0.9421 ± 0.0039**	**0.9152 ± 0.0045**	**29.5**	**8.1**	**118.2**	**121.7**

The bold values provided in the **Table 4** are the results of the method MSCNN-VSS proposed in the paper.

As shown in **Table 4**, the model MSCNN-VSS demonstrates an excellent balance between performance and efficiency. This model achieved the optimal results in segmentation accuracy (PA: 0.9421, mIoU): (0.9152), the number of parameters (29.5M) and computational complexity (8.1G FLOPs) are significantly lower than those of Transformer-based models (such as PD-TR and CMTNet), and its model size (118.2MB) is also much smaller than these models, which demonstrates the advantages of the model. Compared with the benchmark model VM-UNet, which is also part of the VSS series, this model achieved a significant improvement of over 5% in mIoU with only a 17.5% increase in parameters and an 8% increase in computational load. This excellent balance between accuracy and efficiency makes MSCNN-VSS particularly suitable for deployment and application in practical agricultural scenarios with limited computing resources.

Based on the VM-UNet baseline, we progressively integrate four key components: Superpixel segmentation, dilated multi-scale Inception modules, advanced feature fusion mechanisms, and attention mechanisms, to quantitatively evaluate their individual and collective contributions to the final detection performance. This structured ablation study meticulously analyzes improvements in PA, mIoU, and training/inference efficiency, demonstrating that our architectural enhancements achieve significant performance gains while maintaining computational feasibility. The results are shown in **Table 5**.

**Table 5:** The different experimental set and results.

Results Model variant	PA	mIoU	Training time (min)
VM-UNet (Baseline)	0. 8912	0.8423	113.3
+ Superpixel	0.8901	0.8412	104.6
+Dilated Multi-scale Inception	0.9234	0.8765	119.7
+ Feature Fusion	0.9357	0.9016	117.4
+ Attention Mechanism	0. 9368	0.9087	118.9
MSCNN-VSS (Full)	0.9421	0.9152	121.7

The ablation results in **Table 5** illustrate the progressive performance improvements contributed by each component of the MSCNN-VSS architecture. While the superpixel module slightly degrades detection accuracy, it brings notable computational efficiency. The dilated multi-scale Inception module delivers the most substantial gain, significantly increasing both PA and mIoU. Combining feature fusion and attention mechanisms further enhances detection accuracy, with the attention mechanism particularly improving fine-grained detection capability as reflected in the remarkable mIoU improvement. By synergistically combining all components, the complete model achieves the optimal balance between accuracy and efficiency, attaining the highest scores (0.9421 PA, 0.9152 mIoU) with only a 7.4% increase in training time compared to the baseline, thereby validating the effectiveness of the MSCNN-VSS architectural design.

### 3.4 Results analysis

The above experimental results ultimately verified that each component plays a role in improving the model’s accurate disease detection ability. The complete MSCNN-VSS structure achieves the best balance between accuracy and efficiency. As shown in **Table 2**, this model has achieved the most advanced results, with a pixel accuracy of 0.9421 and a mIoU of 0.9152. It is far superior to all comparison methods such as CMTNet (0.8763 PA, 0.8130 mIoU) and the original VM-UNet (0.8912PA, 0.8423 mIoU). This significant performance improvement is achieved while maintaining outstanding training efficiency, requiring 121.7 minutes of training time, which is nearly 50% less than that of CMTNet (254.6 minutes) and only 7.4% more than the VM-UNet baseline (113.3 minutes). The ablation experiment results further verify the effectiveness of each architectural component, particularly highlighting the crucial role of the dilated multi-scale Inception module and attention mechanism in capturing complex pathological features while maintaining computational efficiency. These findings jointly verify that MSCNN-VSS is effective and feasible for crop disease detection through optimal balance accuracy and practicality.

## 4 Conclusion and future work

This paper proposed a novel MSCNN-VSS model to address the challenges of crop disease detection in UAV imagery. The hybrid architecture effectively combines multi-scale CNN, VSS, and attention mechanisms to achieve a superior balance between segmentation accuracy and computational efficiency. Extensive experiments confirmed that our model outperforms several state-of-the-art benchmarks. The model is effective for enabling practical, large-scale crop disease monitoring in precision agriculture, facilitating timely and targeted pest management. However, it has limitations: the dataset is constrained to five crops and only rust diseases, performance under extreme environmental conditions (e.g., heavy rain, dense cloud cover) remains untested, and residual complexity hinders direct deployment on low-power devices. Future work will focus on extending the model to multi-disease classification and developing lightweight versions for edge deployment, as outlined in the Discussion section, explore lightweight versions of the architecture for mobile deployment to handle high-resolution UAV image data.

## Data Availability

The original contributions presented in the study are included in the article/supplementary material. Further inquiries can be directed to the corresponding author.
